# Hypoxia-driven tumor immune escape: mechanisms and therapeutic opportunities

**DOI:** 10.3389/fimmu.2026.1893099

**Published:** 2026-07-01

**Authors:** Hongran Qin, Shuqiang Yang, Jiawei He, Meijia Zhao, Luqian Zhao, Jingjing Wang, Xiulin Jiang, Xiaowen Chen, Xin Xu

**Affiliations:** 1 Department of Nuclear Radiation, Shanghai Pulmonary Hospital, School of Medicine, Tongji University, Shanghai, China; 2 College of Life Science, University of Chinese Academy of Sciences, Beijing, China

**Keywords:** cancer immunotherapy, HIF-1α, hypoxia, immune checkpoints, immune escape, metabolic reprogramming, myeloid-derived suppressor cells, RNA epitranscriptomics

## Abstract

Hypoxia is a common feature of solid tumors and a major driver of tumor immune escape. It arises from abnormal tumor vasculature and rapid tumor growth, leading to persistent or fluctuating oxygen deprivation within the tumor microenvironment. Under hypoxic conditions, hypoxia-inducible factors, especially HIF-1α and HIF-2α, activate transcriptional programs that support tumor survival, angiogenesis, glycolytic metabolism, invasion, and therapy resistance. Beyond these tumor-intrinsic effects, hypoxia also reshapes antitumor immunity by suppressing CD8^+^ T cells, natural killer cells, and dendritic cell antigen presentation, while promoting regulatory T cells, tumor-associated macrophages, and myeloid-derived suppressor cells. These changes establish a strongly immunosuppressive microenvironment and reduce the efficacy of immunotherapy. Hypoxia-driven immune escape is mediated by several interconnected mechanisms. HIF signaling promotes lactate accumulation, acidosis, adenosine signaling, PD-L1 expression, myeloid cell recruitment, and T cell exhaustion. In addition, emerging evidence indicates that RNA modifications, including m6A and ac4C, provide a post-transcriptional regulatory layer that links hypoxia signaling with immune checkpoint regulation, chemokine production, myeloid metabolism, and HIF-1α translation. Therapeutically, targeting hypoxia-related pathways may improve antitumor immunity, but single-agent approaches are often insufficient because hypoxic tumors use multiple overlapping escape mechanisms. Rational combinations involving HIF inhibitors, metabolic intervention, immune checkpoint blockade, cell therapy optimization, and RNA epitranscriptomic targeting may provide more effective strategies. In this review, we summarize how hypoxia coordinates metabolic barriers, immune remodeling, checkpoint activation, and RNA modification-dependent regulation to drive tumor immune escape, and discuss future directions for targeting the hypoxia–metabolism–immune axis in cancer immunotherapy.

## 1 Introduction

Hypoxia is one of the most common features of solid tumors. It develops when rapid tumor growth is not matched by an efficient vascular system. As a result, oxygen delivery becomes insufficient and oxygen diffusion is limited in local tumor regions (1). Tumor hypoxia is not a static condition. It may persist as chronic hypoxia or fluctuate as cyclic hypoxia. Both forms can reshape tumor behavior and therapy response (2). The cellular response to hypoxia is mainly controlled by hypoxia-inducible factors (HIFs). Among them, HIF-1α and HIF-2α are the major transcriptional regulators (2). Under low oxygen conditions, HIF proteins are stabilized, move into the nucleus, and activate transcriptional programs that help tumor cells adapt. These programs include angiogenesis, glycolysis, reduced mitochondrial oxidative phosphorylation, survival signaling, invasion, and metastasis (3). However, hypoxia is not only a tumor cell-intrinsic adaptation mechanism. It also strongly remodels the tumor immune microenvironment (4).

Hypoxia can weaken cytotoxic immune cells, impair antigen presentation, promote immunosuppressive myeloid cells, and increase immune checkpoint expression. In this way, hypoxia connects metabolic stress with immune escape (4). Mechanistically, hypoxia promotes tumor immune evasion through three major layers. First, HIFs directly regulate immune-related genes, including checkpoint molecules and chemokines. Second, hypoxia drives metabolic remodeling, especially glycolysis, lactate accumulation, acidosis, and adenosine signaling. These metabolic changes create a hostile environment for T cells and natural killer (NK) cells. Third, hypoxia changes immune cell composition by suppressing effector cells and expanding regulatory T cells (Tregs), tumor-associated macrophages (TAMs), and myeloid-derived suppressor cells (MDSCs) (4). Recent studies also suggest that hypoxia acts through RNA-level regulation. RNA modifications such as m6A and ac4C can fine-tune HIF signaling, immune checkpoint expression, chemokine production, and myeloid cell metabolism. This adds a post-transcriptional layer to hypoxia-induced immune escape (5).

This mini review summarizes how hypoxia drives tumor immune escape. We focus on HIF signaling, immune cell dysfunction, metabolic checkpoints, RNA modifications, and therapeutic strategies. Rather than simply listing individual findings, we highlight common mechanisms, context-dependent effects, and unresolved questions. We also discuss why targeting hypoxia alone may be insufficient and why future therapies should integrate hypoxia modulation with immunotherapy, metabolic intervention, and RNA epitranscriptomic targeting. Importantly, this review also critically discusses several unresolved issues in the field, including the context-dependent role of HIF signaling in different immune cell types, the dual effects of metabolic targeting on tumor and immune cells, and the limited causal evidence linking RNA modifications to hypoxia-driven immune escape. We further summarize these controversies in a dedicated synthesis table and propose conceptual models to explain why hypoxia-targeted interventions show variable outcomes across tumor contexts. In addition to canonical HIF-dependent transcriptional programs, we also discuss noncanonical hypoxia-associated stress responses, including UPR/ER stress, ROS signaling, mitochondrial stress adaptation, and autophagy, which may independently or cooperatively shape immune escape.

## 2 Canonical and noncanonical hypoxia signaling in tumor immune escape

The core hypoxia signaling network is controlled by HIFs. HIF-1α and HIF-2α share structural similarity but also have distinct functions (5). Under normal oxygen levels, HIF-α subunits are hydroxylated by prolyl hydroxylase domain enzymes and then recognized by the von Hippel–Lindau ubiquitin–proteasome system for degradation (5). Under hypoxia, this degradation pathway is inhibited. HIF-α proteins accumulate, translocate into the nucleus, dimerize with HIF-β, bind hypoxia response elements, and activate broad transcriptional programs (6). This response is essential for cell survival under oxygen shortage. In cancer, however, the same adaptive program becomes a driver of progression and immune resistance. HIF signaling links metabolism, vascular remodeling, immune suppression, and therapy resistance. It therefore functions less like a single pathway and more like an organizing hub of the hypoxic tumor ecosystem.

### 2.1 HIF-driven metabolic reprogramming

One of the best-characterized functions of HIF signaling is metabolic reprogramming. HIF-1α shifts tumor metabolism from mitochondrial oxidative phosphorylation toward glycolysis-dependent energy production (6, 7). It directly induces glucose transporters and glycolytic enzymes, including GLUT1, HK2, PFK1, and LDHA (2). This increases glucose uptake and lactate production. This shift supports tumor cell survival under hypoxia, but it also reshapes the immune microenvironment. Lactate accumulation and extracellular acidification suppress CD8+ T cell and NK cell function and reduce antigen presentation efficiency (2). Therefore, HIF-driven glycolysis is not only a metabolic adaptation. It becomes an immune barrier. A critical point is that the Warburg-like phenotype in hypoxic tumors does not simply provide ATP. It also changes nutrient availability, pH, redox state, and immune cell metabolism. Effector T cells also require glycolysis for activation (7). When tumor cells dominate glucose consumption and produce lactate, T cells become metabolically disadvantaged. This explains why hypoxia and glycolysis often correlate with poor immune infiltration and resistance to immunotherapy.

### 2.2 Lactate as an immunoregulatory metabolite

Lactate was once viewed as a metabolic waste product. It is now recognized as an active immunoregulatory molecule (8) High lactate levels promote M2-like TAM polarization and enhance MDSC suppressive function (8) Lactate also directly inhibits T cell proliferation and reduces cytokine production, including IFN-γ (8) The acidic microenvironment caused by lactate further impairs T cell metabolic adaptation. T cells lose glycolytic capacity, develop energy imbalance, and gradually become dysfunctional or exhausted (8). Thus, lactate acts at several levels: it weakens effector cells, strengthens suppressive myeloid cells, and stabilizes an immunosuppressive niche. This has therapeutic implications. Targeting lactate production or transport may improve immunotherapy, but complete inhibition of glycolysis may also harm activated immune cells. Therefore, effective metabolic targeting should aim to selectively reduce tumor-driven lactate barriers while preserving immune cell function.

### 2.3 HIF-driven immunosuppressive gene programs

HIF signaling also directly controls immune-related genes. HIF-1α can upregulate PD-L1 directly or indirectly, increasing checkpoint-mediated T cell suppression (9). HIF signaling also regulates VEGF, CCL2, and CXCL12, which recruit suppressive myeloid cells and inhibit dendritic cell maturation (9). At the adaptive immune level, HIF signaling can promote Treg stability while reducing CD8+ T cell cytotoxicity and memory formation (10). These effects shift the tumor from an immune-recognizable state to an immune-evasive state. Therefore, HIF is not only a hypoxia-response factor. It is also a transcriptional organizer of immune suppression (10). However, HIF biology is context dependent. In some immune cells, HIF-1α may support glycolytic adaptation and effector function. In others, it may drive exhaustion or suppressive phenotypes. This dual role is important. It means that systemic HIF inhibition may have different effects on tumor cells and immune cells. Future therapeutic strategies must consider cell type, timing, and oxygen context.

### 2.4 HIF-independent and noncanonical hypoxia stress responses

Although HIF signaling is the best-characterized hypoxia-response pathway, tumor hypoxia also activates several HIF-independent or partially HIF-dependent stress programs. These noncanonical mechanisms are important because they can influence immune escape even when canonical HIF transcriptional activity is inhibited (11). One major pathway is the unfolded protein response (UPR) (11). Hypoxia disrupts protein folding in the endoplasmic reticulum and activates PERK, IRE1α, and ATF6 signaling (11). The PERK–eIF2α–ATF4 axis can promote metabolic adaptation, antioxidant responses, autophagy, and immune dysfunction (12). In immune cells, chronic ATF4 activation has been linked to mitochondrial stress, oxidative imbalance, and CD8+ T-cell dysfunction (13). In tumor cells and antigen-presenting cells, ER stress may reduce antigen processing, impair MHC-I presentation, and weaken T-cell priming (14).

Hypoxia also reshapes redox and mitochondrial homeostasis (15). Oxygen deprivation and reoxygenation can increase mitochondrial ROS, alter electron transport chain activity, and induce mitochondrial stress adaptation (15). Moderate ROS may support adaptive signaling, whereas sustained ROS damages mitochondria, promotes inflammatory stress responses, and contributes to T-cell exhaustion or tumor cell survival programs (15). In parallel, hypoxia can activate autophagy and mitophagy. These processes may help tumor cells survive nutrient and oxygen deprivation, but they can also reduce antigen availability by degrading antigen-processing machinery or limiting immunogenic cell death. Therefore, autophagy has a dual role: it may support stress tolerance in tumor cells while also modulating antigen presentation and immune recognition (15).

These mechanisms indicate that hypoxia should not be viewed solely as a HIF transcriptional program. Instead, it represents an integrated stress state involving HIF signaling, UPR/ER stress, ROS signaling, mitochondrial remodeling, autophagy, and metabolic adaptation. This broader view may explain why HIF inhibition alone is often insufficient and why rational therapeutic strategies may need to combine HIF targeting with interventions that modulate ER stress, mitochondrial fitness, redox balance, or autophagy.

### 2.5 Controversies and open questions in hypoxia–HIF biology

**Figure 1:**
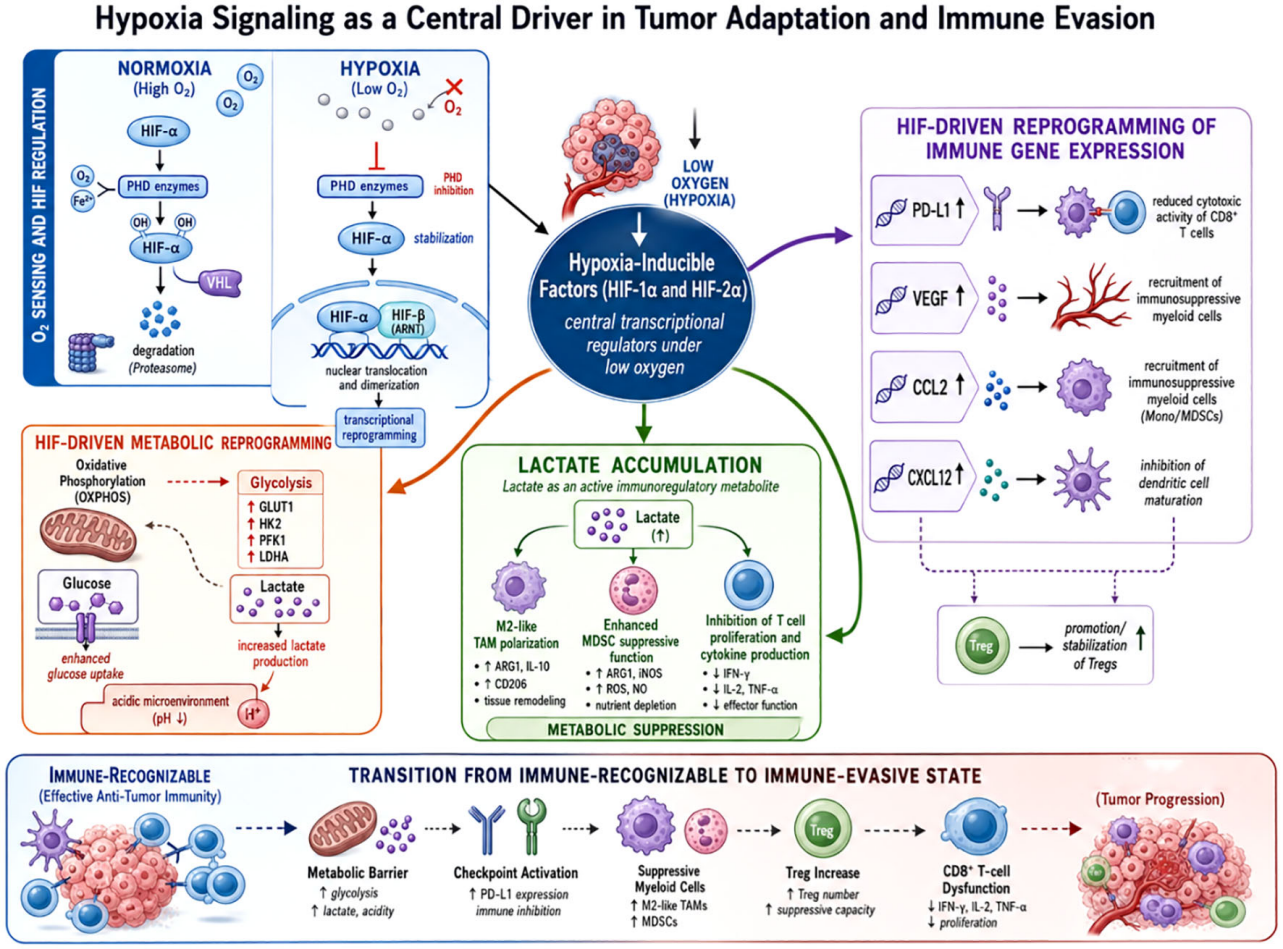
Hypoxia signaling as a central driver in tumor adaptation and immune evasion. Under normoxic conditions, HIF-α is hydroxylated by PHD enzymes, recognized by VHL, and degraded through the proteasome. Under hypoxia, HIF-α is stabilized, translocates into the nucleus, dimerizes with HIF-β, and activates transcriptional reprogramming. HIF signaling promotes glycolytic metabolism by upregulating GLUT1, HK2, PFK1, and LDHA, leading to increased glucose uptake, lactate production, and extracellular acidification. Lactate further promotes M2-like TAM polarization, enhances MDSC suppressive function, and inhibits T-cell proliferation and cytokine production. In parallel, HIF-driven immune gene regulation increases PD-L1, VEGF, CCL2, and CXCL12 expression, which supports immune checkpoint activation, suppressive myeloid cell recruitment, Treg stabilization, dendritic cell dysfunction, and CD8^+^ T-cell impairment. Together, these mechanisms drive the transition from an immune-recognizable tumor state to an immune-evasive state.

Although HIF signaling is widely viewed as a driver of tumor immune escape, its biological effects are not uniformly immunosuppressive. A major unresolved issue is whether HIF inhibition should be applied broadly or in a cell type-specific manner. In tumor cells and suppressive myeloid cells, HIF-1α and HIF-2α often promote glycolysis, lactate production, VEGF signaling, PD-L1 expression, and recruitment of immunosuppressive cells. In contrast, in activated CD8+ T cells and NK cells, HIF-1α-dependent glycolysis may support short-term effector function and adaptation to oxygen deprivation (16). Therefore, systemic HIF blockade may simultaneously suppress tumor adaptation and impair immune cell fitness. Another open question is why hypoxia-targeted therapies show heterogeneous outcomes across tumor types. Possible explanations include differences in chronic versus cyclic hypoxia, tumor vascular architecture, baseline immune infiltration, dominant suppressive cell populations, and the relative contribution of HIF-dependent versus HIF-independent stress pathways. Future studies should distinguish tumor cell-intrinsic HIF programs from immune cell-intrinsic HIF functions and should define when hypoxia acts primarily as a metabolic barrier, a checkpoint-inducing signal, or a myeloid-recruiting cue. To clarify how hypoxia coordinates tumor adaptation and immune escape, we first summarize the central role of HIF signaling in metabolic reprogramming, lactate accumulation, immune checkpoint activation, and the transition toward an immune-evasive tumor state (**Figure 1**).

## 3 Hypoxia shapes an immunosuppressive microenvironment

**Figure 2:**
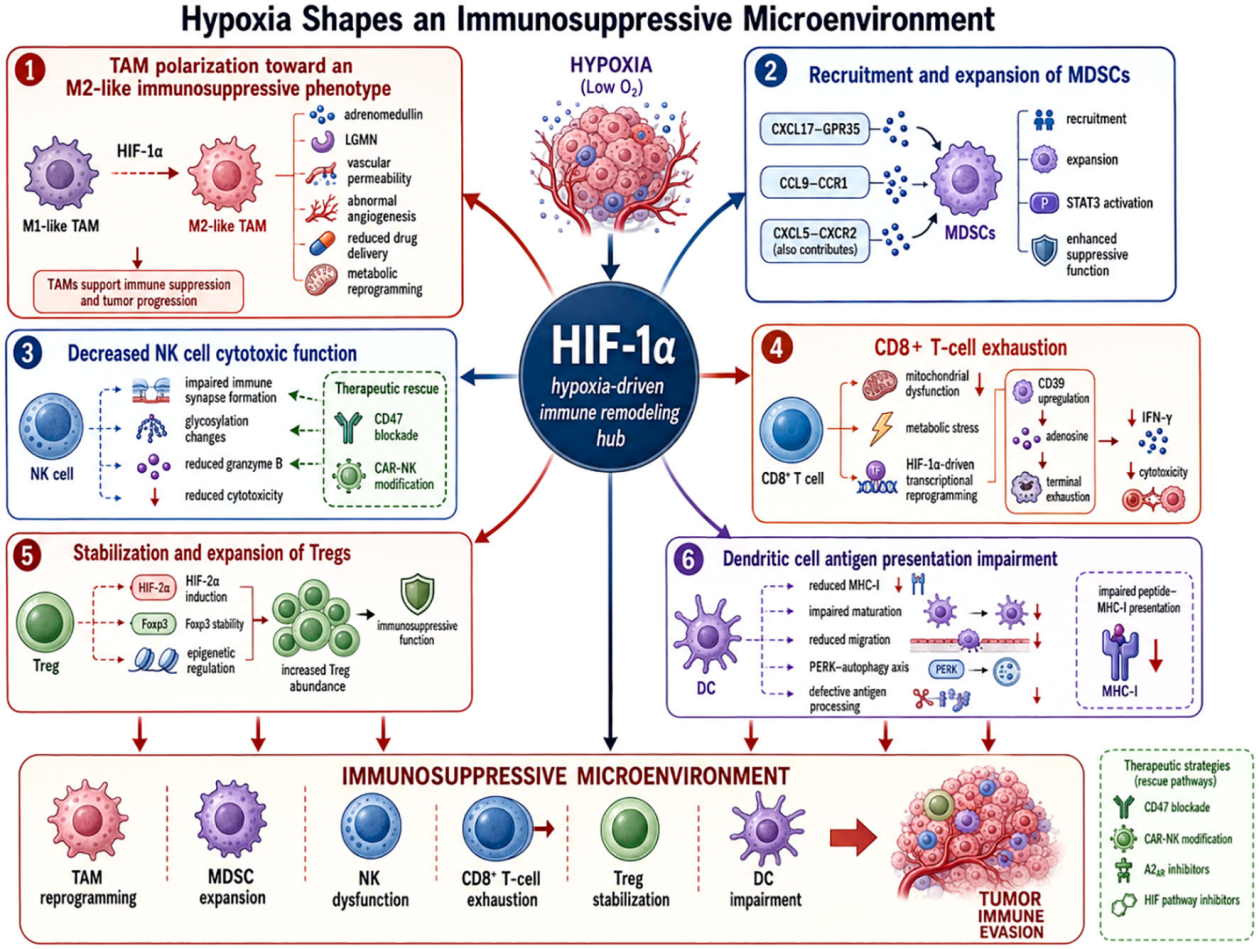
Hypoxia shapes an immunosuppressive microenvironment. Hypoxia activates HIF-1α-centered immune remodeling and suppresses antitumor immunity through multiple immune cell populations. In innate immunity, hypoxia promotes TAM polarization toward an M2-like immunosuppressive phenotype, partly through factors such as adrenomedullin and LGMN, which support vascular dysfunction, abnormal angiogenesis, metabolic reprogramming, and reduced drug delivery. Hypoxia also recruits and expands MDSCs through chemokine axes such as CXCL17–GPR35, CCL9–CCR1, and CXCL5–CXCR2, leading to STAT3 activation and enhanced suppressive function. In NK cells, hypoxia impairs immune synapse formation, alters glycosylation-dependent recognition, reduces granzyme B release, and weakens cytotoxicity. In adaptive immunity, hypoxia promotes CD8^+^ T-cell exhaustion through mitochondrial dysfunction, metabolic stress, HIF-1α-driven transcriptional changes, and CD39–adenosine signaling. It also stabilizes and expands Tregs through HIF-2α, Foxp3 regulation, and epigenetic mechanisms. In dendritic cells, hypoxia impairs maturation, migration, MHC-I expression, and antigen processing through pathways such as PERK–autophagy. These coordinated effects lead to TAM reprogramming, MDSC expansion, NK dysfunction, CD8^+^ T-cell exhaustion, Treg stabilization, and DC impairment, ultimately promoting tumor immune evasion.

Because hypoxia affects both innate and adaptive immune compartments, we next illustrate how HIF-1α-centered signaling remodels major immune cell populations and establishes an immunosuppressive tumor microenvironment (**Figure 2**).

### 3.1 Innate immune suppression

#### 3.1.1 TAM polarization toward immunosuppressive states

TAMs are highly enriched in hypoxic tumor regions and are strongly shaped by HIF-dependent signaling (17). In many tumors, hypoxia pushes TAMs toward M2-like or otherwise immunosuppressive states. These macrophages support tumor progression through immune suppression, angiogenesis, tissue remodeling, and metabolic cooperation with tumor cells. Single-cell and spatial transcriptomic studies show that monocyte-derived TAMs are spatially enriched in perinecrotic hypoxic niches in glioblastoma (18). These TAMs express hypoxia-responsive programs and acquire immunosuppressive features. Hypoxic TAMs can secrete adrenomedullin, which disrupts endothelial junctions, increases vascular permeability, and promotes abnormal angiogenesis (18). This links hypoxic immune suppression with vascular dysfunction and poor drug delivery. Hypoxic TAMs can also regulate tumor metabolism. In gastric cancer, M2-like TAM-derived exosomes carry lncRNA MALAT1 into tumor cells. MALAT1 suppresses β-catenin degradation and enhances HIF-1α signaling, promoting glycolysis, tumor growth, metastasis, and drug resistance (19). This example shows that TAMs are not passive responders to hypoxia. They can actively reinforce hypoxic metabolic programs in tumor cells.

At the molecular level, hypoxia controls immunosuppressive molecules in TAMs. In glioblastoma, legumain (LGMN) is enriched in TAMs and regulated by HIF-1α (17). LGMN activates the GSK-3β–STAT3 axis, promotes immunosuppressive TAM polarization, and inhibits CD8+ T cell-mediated immunity (20). Inhibition of HIF-1α or LGMN restores antitumor responses and improves PD-1 blockade (20). Hypoxia also alters TAM spatial distribution and interaction networks. During glioma progression, TAMs shift from perivascular regions toward hypoxic niches. In these regions, TAMs and CD8+ T cells can become co-localized but functionally trapped through signals such as CCL8 and IL-1β (21).This creates a pseudopalisading hypoxic niche where immune cells are present but ineffective. Inflammatory feedback further strengthens TAM-driven immune suppression. SPHK1+ TAMs are regulated by HIF-1α and NF-κB. SPHK1 promotes S1P production, activates the NLRP3 inflammasome, and increases IL-1β secretion (22). IL-1β then induces chemokines and ADAM17, promoting further TAM infiltration and CD8+ T cell exhaustion (22).Hypoxia can also influence TAM cell-cycle status through HIF-2α, increasing CDK1-related programs and functional plasticity (23). Metabolic crosstalk adds another layer. In hepatocellular carcinoma, loss of SLC27A2 redistributes long-chain fatty acids between tumor and immune cells. Lipid uptake by TAMs activates PPARγ signaling and enriches SPP1+ immunosuppressive TAMs (24). Similarly, IL-4/STAT6 signaling can induce Siglec-G/10 and interact with HIF-1α to sustain TAM immunosuppressive function (25). Overall, hypoxia drives TAM immunosuppression through transcriptional regulation, metabolic exchange, exosome-mediated communication, inflammatory feedback, and spatial remodeling. A key conclusion is that hypoxic TAMs are not one uniform M2 population. They contain multiple functional states, and these states may require different therapeutic strategies.

#### 3.1.2 Recruitment and expansion of MDSCs

Hypoxia is also a strong driver of MDSC recruitment, expansion, and suppressive activity (26, 27). MDSCs are important because they suppress T cells, shape macrophage polarization, and reduce immunotherapy efficacy. In liver transplantation and ischemia-reperfusion injury models, MDSC-like populations increase in peripheral blood and liver tissue after surgery (28). Hypoxia/reoxygenation activates YAP/TEAD1 signaling in hepatic endothelial cells, which induces CXCL17. CXCL17 then binds GPR35 and promotes MDSC recruitment (28). These MDSCs suppress M1 macrophage polarization through STAT3 activation and reduce inflammatory injury (28). Although this is not a tumor model, it shows how hypoxia-related tissue stress can recruit immunosuppressive myeloid cells. In tumors, hypoxia can promote MDSC expansion through adaptive therapy-related feedback. In pancreatic ductal adenocarcinoma, a hypoxia-activated prodrug and VEGFR2 blockade improve vascular structure and increase tumor cell death, but do not fully reduce hypoxia or improve immune checkpoint blockade (29). Instead, tumor cells upregulate CCL9, which recruits granulocytic MDSCs through CCR1 (29).]. Blocking CCL9/CCR1 or depleting Ly6G+ cells restores checkpoint therapy efficacy (29). This example is important because it shows that partial vascular or hypoxia modulation may trigger compensatory myeloid suppression.

Hypoxia also recruits MDSCs through stromal remodeling. In colorectal cancer liver metastasis resistant to anti-angiogenic therapy, bevacizumab-induced hypoxia increases FGFBP1 in tumor cells. This activates FGF2/FGFR1/ERK1/2/EGR1 signaling in hepatic stellate cells and induces FAPα expression (30). FAPα+ stellate cells then secrete CXCL5, recruiting MDSCs through CXCR2 and promoting EMT (30). This vessel co-option–MDSC axis illustrates how hypoxia can link therapy resistance, stromal remodeling, and immune suppression. MDSC function is also metabolically controlled by hypoxia. In Staphylococcus aureus biofilm infection, granulocytic MDSCs show enhanced glycolysis and HIF-1α-dependent metabolism. Blocking HIF-1α or glycolysis reduces their suppressive function (29). In fetal intrauterine hypoxia-related disease models, PMN-MDSCs increase lactate production and suppress microglial activation through the HIF-1α–lactate axis (31). These non-cancer models reinforce the principle that hypoxia-driven glycolysis helps maintain MDSC suppressive activity. Together, these findings show that hypoxia promotes MDSCs through chemokine recruitment, stromal activation, and metabolic reprogramming. From a therapeutic view, simply reducing tumor hypoxia may not be enough if compensatory chemokine pathways remain active. Combined targeting of hypoxia and MDSC recruitment axes may be required.

#### 3.1.3 Decreased NK cell cytotoxic function

NK cells are important innate immune effectors, but hypoxia often weakens their antitumor function (4). Hypoxia can impair NK cell infiltration, survival, target recognition, immune synapse formation, and cytotoxic molecule release (4). In colorectal cancer models, NK cells exposed to tumor organoids acquire hypoxia- and TGF-β-associated transcriptional signatures similar to NK cells in patient tumors (29). HIF-related programs cooperate with TGF-β signaling to shift NK cells away from highly cytotoxic states toward dysfunctional or tissue-resident-like states (29). Hypoxia can also change tumor cell recognition by NK cells. Under hypoxic stress, tumor cells upregulate Siglec-7/9-associated sialylated ligands (32). NK cell Siglec-7/9 expression may not change, but tumor cell glycosylation is remodeled. This weakens immune synapse stability and reduces granzyme B release (32). Sialic acid-mimicking nanoparticles that block Siglec interactions can restore NK cell synapse formation and cytotoxicity (32). This suggests that hypoxia-induced glycosylation changes are an underappreciated immune escape mechanism.

Checkpoint pathways also interact with NK cell recovery. In hepatocellular carcinoma, CD47 blockade activates a CD103+ DC–NK cell axis (33). CD103+ DCs produce IL-12 and CXCL9, recruit NK cells, and increase NKG2D, granzyme B, IFN-γ, and TNF-α (33).This effect depends on cGAS–STING signaling, indicating that NK restoration under hypoxia may require innate immune amplification. Engineered NK cell therapies may overcome some hypoxia-related barriers. HER1-targeted CAR-NK cells expressing catalase reduce oxidative stress and local hypoxia in triple-negative breast cancer models, improving NK cell survival, infiltration, and cytotoxicity (34). This strategy also suppresses distant metastasis (34). The role of HIF-1α in NK cells is complex. In primary NK cells, hypoxia may fail to stabilize HIF-1α and instead reduce cytotoxicity. Under IL-2 stimulation or PI3K–mTOR activation, HIF-1α can be stabilized and support metabolic adaptation and IFN-γ production (35). Thus, HIF-1α can either support or impair NK activity depending on activation status and metabolic context. Overall, hypoxia suppresses NK cells through metabolic inhibition, TGF-β-related reprogramming, altered glycosylation, and immune synapse disruption. Therapeutic recovery may require combined strategies that improve oxygen balance, block inhibitory recognition pathways, and enhance innate immune activation.

### 3.2 Adaptive immune dysfunction

#### 3.2.1 CD8+ T cell exhaustion

CD8+ T cell exhaustion is a major feature of tumor immune escape. Exhausted T cells express inhibitory receptors, produce fewer cytokines, and show reduced cytotoxicity (36)]. Exhaustion is not a single terminal state. It is a dynamic hierarchy that includes progenitor exhausted T cells (Tpex) and terminal exhausted T cells (36) Hypoxia influences the transition between these states. Metabolic stress is a key driver of exhaustion. Mitochondrial dysfunction causes redox imbalance and disrupts HIF-1α degradation, leading to abnormal HIF-1α stabilization and transcriptional reprogramming (37). This promotes the differentiation of Tpex cells toward terminal exhaustion and weakens sustained antitumor immunity (37). Metabolic engineering of CAR-T cells can improve Tpex-like features and long-term persistence, supporting the importance of the metabolism–exhaustion axis (37).

P4HA1 is another regulator of T cell metabolism under hypoxia. It is increased in tumor-draining lymph nodes and hypoxic tumor tissues (38). In CD8+ T cells, P4HA1 localizes to mitochondria and disrupts the TCA cycle, altering α-ketoglutarate and succinate metabolism (38). This suppresses TCF1+ progenitor T cell expansion and accelerates exhaustion (38). Targeting P4HA1 restores systemic antitumor immunity. Hypoxia also promotes terminal exhaustion through CD39–adenosine signaling. Terminal exhausted CD8+ T cells can acquire Treg-like suppressive features and inhibit other T cells through CD39-mediated adenosine production (39). CD39 is regulated by hypoxia, and inhibition of hypoxic signaling reduces the suppressive capacity of terminal exhausted T cells (39). Integrated stress responses further contribute to exhaustion. Hypoxia activates the ATF4 axis through the integrated stress response, promoting metabolic polarization, mitochondrial oxidative stress, and CD8+ T cell death (13). Sustained HIF-1α-related metabolic stress and ATF4 signaling can induce terminal exhaustion-like programs even without continuous TCR stimulation (13). This indicates that hypoxia can drive exhaustion independently of antigen stimulation intensity. Hypoxia can also act indirectly through tumor and myeloid cells. In hepatocellular carcinoma, HIF-1α induces RCOR2, which enhances glycolysis, tumor progression, M2 macrophage polarization, and CD8+ T cell exhaustion (40). In high-grade serous ovarian cancer, hypoxia-driven VEGFA–EPHB2 signaling promotes macrophage state transitions and accelerates CD8+ T cell progression toward terminal exhaustion (41).

However, HIF-1α is not always harmful to CD8+ T cells. In some contexts, HIF-1α-regulated glycolysis supports effector function. For example, GP73 deficiency impairs glycolysis under hypoxia and accelerates T cell exhaustion, while GP73 restoration stabilizes HIF-1α and improves cytotoxicity (42). This suggests that the effect of HIF-1α depends on whether it supports adaptive metabolism or drives chronic stress. Overall, hypoxia drives CD8+ T cell exhaustion through mitochondrial dysfunction, HIF-1α signaling, ATF4 activation, CD39–adenosine production, and myeloid remodeling. A key therapeutic challenge is to reduce hypoxia-induced stress while preserving the metabolic programs needed for T cell effector activity. Beyond HIF stabilization, mitochondrial stress and ROS signaling are central mediators of hypoxia-induced T-cell dysfunction. Chronic oxygen deprivation can impair electron transport chain activity, increase mitochondrial ROS, and reduce mitochondrial fitness (43). These changes compromise cytokine production, cytotoxic granule release, and long-term persistence of CD8+ T cells. Importantly, ROS signaling is dose- and context-dependent. Moderate ROS may function as an activation-associated signal, whereas sustained ROS accumulation promotes oxidative damage, metabolic failure, and exhaustion. Therefore, therapeutic strategies aimed at improving T-cell function in hypoxic tumors should consider mitochondrial protection and redox balance rather than focusing only on HIF inhibition.

#### 3.2.2 Stabilization and expansion of Tregs

Hypoxia promotes Treg recruitment, stability, and suppressive function (44). Tregs are important in hypoxic tumors because they limit effector T cell activity and support immune tolerance. Epigenetic regulation contributes to Treg stability. HDAC dysregulation is associated with altered Foxp3+ Treg function (45). HDAC inhibitors such as SAHA can restore FOXP3 expression and promote Treg reprogramming in disease models (45). In cancer, however, the therapeutic goal may not always be to increase Tregs. This highlights the context-specific nature of epigenetic modulation. HIF-2α is a key regulator of immunosuppressive environments in glioblastoma. It promotes the accumulation and suppressive function of Tregs and TAMs (46). HIF-2α inhibition with PT2385 reduces Tregs and synergizes with PD-1 or TIM-3 blockade, enhancing antitumor immunity (46). Single-cell studies further show that HIF-2α inhibition shifts the TME from suppressive to more immunostimulatory states.

Hypoxia normalization can also reshape Treg dominance. In colorectal cancer models, combined chemotherapy, anti-angiogenic therapy, and PD-1 blockade improve vascular function and reduce HIF-1α expression (47). This activates cGAS–STING signaling and increases dendritic cells, CD8+ T cells, and NK cells without increasing Tregs or M2 macrophages (47). These findings suggest that reducing hypoxia can restore immune balance. Spatial studies show that Tregs often accumulate in hypoxic niches. In hepatocellular carcinoma, hypoxic regions contain high levels of Tregs and suppressive myeloid cells but fewer granzyme B+ CD8+ T cells (48). Hypoxia-induced chemokines such as CCL20 and CXCL5 recruit Tregs and cDC2 subsets while reducing antigen presentation, including HLA-DR expression (48). Treg–cDC2 interactions then further impair antigen presentation. Overall, hypoxia stabilizes Treg-mediated suppression through HIF signaling, chemokine recruitment, epigenetic regulation, and impaired antigen presentation. Therapeutically, this supports combined strategies that target hypoxia, Tregs, and suppressive myeloid cells rather than Tregs alone.

#### 3.2.3 Impaired dendritic cell antigen presentation

Dendritic cells are required for T cell priming, but hypoxia can impair their antigen presentation capacity (49, 50). In solid tumors, hypoxia reduces DC maturation, migration, antigen processing, and MHC expression. This weakens T cell priming and makes tumors less visible to the immune system (50). One important mechanism is the reduction of MHC-I expression and immunopeptidome diversity. Hypoxia decreases surface antigen presentation through the PERK arm of the unfolded protein response (50). The PERK–autophagy axis promotes degradation of antigen-processing machinery and reduces peptide loading efficiency (49). This mechanism is particularly important because it links hypoxia to immune escape through a pathway that is not limited to HIF-dependent transcription. ER stress can alter protein folding, peptide processing, and the availability of tumor antigens for MHC loading (51). In parallel, hypoxia-induced autophagy may degrade components of the antigen-processing machinery or reduce the pool of immunogenic peptides (51). As a result, tumors may become less visible to T cells even when immune checkpoints are blocked. These findings suggest that restoring antigen presentation may require targeting ER stress or autophagy in addition to HIF signaling.

Immunopeptidomic analyses confirm that hypoxia reduces the diversity of presented antigens. Inhibiting autophagy or restoring mitochondrial metabolism can rescue MHC-I expression and antigen presentation (49). Hypoxia also affects MHC-II-related antigen presentation through HIF-1α-dependent myeloid reprogramming. In transplant rejection models, the TLR4–HIF-1α axis expands inflammatory macrophages but suppresses MHC-II+ antigen-presenting cells (52, 53). This shifts T cell composition toward fewer CD8+ and Th1 cells and more Tregs and Th2 cells (53). In tumors, similar hypoxia-driven pathways may reduce DC maturation and lymph node migration (50).

From a therapeutic perspective, antigen presentation impairment is important because it may limit the success of checkpoint blockade. If tumor antigens are poorly presented, releasing PD-1 inhibition alone may not restore immunity. Strategies that improve mitochondrial function, reduce hypoxia, block autophagy-driven antigen loss, or activate innate immune sensing may increase antigen visibility (54). Overall, hypoxia can make tumors “antigen invisible” by reducing MHC-I, impairing peptide loading, disrupting MHC-II+ APC function, and inducing metabolic or ER stress. This mechanism is reversible in some models and represents a key target for improving immunotherapy.

### 3.3 Context-dependent immune outcomes of hypoxia

The immune consequences of hypoxia vary substantially across cell types and tumor contexts. In macrophages and MDSCs, hypoxia generally promotes suppressive differentiation, glycolytic metabolism, chemokine production, and resistance to immunotherapy (55). In CD8+ T cells and NK cells, however, the outcome is more complex (56). Acute or moderate HIF activation may support metabolic adaptation, whereas chronic hypoxia, nutrient deprivation, acidosis, and mitochondrial stress tend to drive exhaustion, cytotoxic dysfunction, or cell death. Dendritic cells are affected mainly through impaired antigen processing, reduced MHC expression, and altered maturation (57). These differences suggest that hypoxia should not be interpreted as a single uniform immunosuppressive signal. Instead, hypoxia functions as a context-dependent ecological pressure. The final immune outcome depends on oxygen dynamics, nutrient competition, lactate concentration, inflammatory signals, tumor type, treatment history, and the spatial relationship between tumor cells and immune cells. This may explain why some hypoxia-modulating strategies enhance immunotherapy in certain models but show limited efficacy in others.

## 4 Immune checkpoints and metabolic barriers

Hypoxia regulates immune escape not only by changing immune cell composition but also by directly activating immune checkpoint pathways. HIF-1α connects hypoxia, metabolism, and PD-1/PD-L1 signaling, creating a coordinated metabolism–checkpoint suppression axis (58). PD-L1 is one of the best-characterized HIF-regulated immune checkpoint molecules. In glioma, HIF-1α binds the CD274 promoter and directly activates PD-L1 transcription under hypoxia (9) Blocking HIF-1α or PD-L1 improves DC activation and CD8+ T cell function. Combined HIF-1α inhibition and PD-L1 blockade show stronger antitumor effects *in vivo* (9) This supports the idea that hypoxia can act upstream of immune checkpoint resistance. Lactate metabolism further strengthens PD-L1 regulation. Lactate stabilizes HIF-1α through MCT4-dependent mechanisms and increases PD-L1 expression while suppressing anti-angiogenic immune regulators such as Sema3A (59). Lactate-induced histone lactylation can also enhance HIF1A transcriptional activity, maintaining high PD-L1 expression and forming a metabolism–epigenetics–checkpoint feedback loop (59).

In myeloid cells, hypoxia also induces checkpoint-related reprogramming. In hepatocellular carcinoma, TAMs show high glycolytic activity and express PD-L1 in a HIF-1α-dependent manner (60). PKM2, a glycolytic enzyme regulated by HIF-1α, promotes both inflammatory features and PD-L1 expression (61). These macrophages may show mixed phenotypes but often contribute to immune suppression. In tumor cells, GJB2 can activate NF-κB and the HIF-1α/GLUT1/PD-L1 axis, reinforcing glycolysis-dependent immune escape (62). The spatial and tissue-specific effects of HIF signaling are also important. HIF-1α inhibition can reduce PD-L1 in tumor tissues and restore T cell function, while normal tissues may maintain PD-L1 through IFN-γ-dependent mechanisms (63). This suggests a possible therapeutic window, although this needs careful validation. Immune checkpoints also regulate metabolism beyond the tumor setting. For example, PD-1 signaling can suppress DRP1, impair mitochondrial autophagy, increase ROS, and promote senescence-associated secretory phenotypes in inflammatory disease (64). Endothelial HIF-1α can activate DGKG, enhance TGF-β signaling, and promote Treg differentiation (65).These findings support a broader concept: checkpoint molecules are not only immune brakes. They also participate in metabolic and tissue-regulatory circuits.

## 5 RNA modification regulation in hypoxia-induced tumor immune escape

Hypoxia drives immune escape through transcriptional and metabolic mechanisms, but recent evidence adds a third layer: epitranscriptomic regulation (61). RNA modifications, especially m6A and ac4C, are dynamic under hypoxia and can regulate RNA stability, translation, and immune signaling. This allows hypoxic tumors to fine-tune immune escape after transcription.

### 5.1 m6A-mediated immune escape

m6A can regulate hypoxia-induced immune escape through long non-coding RNAs, chemokines, and myeloid metabolism. In pancreatic cancer, HIF-1α activates lncRNA NNT-AS1, and METTL3–HuR-mediated m6A modification increases NNT-AS1 stability (66). This enhances ITGB1 expression and promotes immune escape by suppressing CD8+ T cell function (66).This defines a hypoxia–HIF–lncRNA–m6A–immune escape axis. The m6A eraser ALKBH5 also contributes to immune suppression under hypoxia. In glioblastoma, hypoxia induces ALKBH5, which stabilizes lncRNA NEAT1 by removing m6A marks (67). NEAT1 promotes paraspeckle formation and changes the nuclear distribution of SFPQ, releasing repression of the CXCL8 promoter (67). CXCL8 then recruits TAMs and promotes immune suppression. This example shows that demethylation can be as important as methylation in hypoxic immune escape. In hepatocellular carcinoma, hypoxia activates a HIF-1α–YTHDF2–PFKL axis in MDSCs (55). HIF-1α upregulates YTHDF2, which stabilizes PFKL mRNA. This increases glycolysis and lactate production, strengthens MDSC suppressive activity, inhibits CD8+ T cell function, and supports tumor stemness (55). Here, m6A reading connects hypoxia to myeloid metabolic suppression. These examples show that m6A does not act through one uniform mechanism. METTL3 may stabilize lncRNAs, ALKBH5 may remodel nuclear RNA architecture, and YTHDF2 may rewire metabolism in MDSCs. The common theme is that m6A regulators help hypoxic tumors convert stress signals into durable immune-suppressive programs.

### 5.2 ac4C-mediated translational control

ac4C is another RNA modification involved in hypoxia adaptation. NAT10, a major ac4C writer, enhances HIF1A mRNA translation in hypoxic tumors, increasing HIF-1α protein levels (68). This amplifies lactate production and PD-L1 expression, creating a positive feedback loop between RNA modification and hypoxia signaling (68). NAT10 inhibition reduces HIF-1α protein levels, decreases intratumoral lactate gradients, suppresses PD-L1-mediated immune inhibition, restores CD8+ T cell function, and improves checkpoint therapy response (68). This mechanism is important because it shows that RNA modification can regulate hypoxia not only downstream of HIF but also upstream of HIF protein production. Overall, RNA modifications represent a regulatory layer that connects hypoxia, RNA fate, metabolism, and immune escape. However, this field is still early. Many studies identify associations between RNA modification enzymes and immune phenotypes, but direct site-specific causal evidence is still limited. Future work should identify which modified RNA sites are necessary for hypoxia-induced immune escape and whether they can be targeted safely.

Collectively, these findings indicate that hypoxia-driven immune escape cannot be explained by a single pathway or cell type. Instead, hypoxia generates a context-dependent immune-suppressive network involving HIF signaling, metabolic stress, myeloid remodeling, lymphocyte dysfunction, impaired antigen presentation, and RNA epitranscriptomic regulation. To provide a comparative overview of these interconnected mechanisms and to highlight key controversies and therapeutic implications, we summarize the major context-dependent features of hypoxia-driven tumor immune escape in **Table 1**.

**Table 1:** Context-dependent mechanisms, controversies, and therapeutic implications of hypoxia-driven tumor immune escape.

Hypoxia-related mechanism	Major immune effect	Context-dependent issue/controversy	Therapeutic implication	Ref
HIF-1α/HIF-2α signaling	Promotes glycolysis, VEGF, PD-L1, myeloid recruitment	HIF may suppress antitumor immunity in tumor/myeloid cells but support metabolic adaptation in activated T/NK cells	Cell type-specific or timed HIF targeting may be preferable to broad inhibition	(84, 85)
Lactate/acidosis	Suppresses CD8+ T cells and NK cells; promotes TAM/MDSC function	Blocking glycolysis may also impair activated effector T cells	Target lactate transport or tumor-selective lactate production rather than global glycolysis	(86, 87)
TAM remodeling	Promotes angiogenesis, immune suppression, tissue remodeling	Hypoxic TAMs are heterogeneous and not simply M2-like	Combine hypoxia targeting with TAM reprogramming strategies	(18, 21)
MDSC recruitment	Suppresses T-cell function and limits checkpoint blockade	Partial vascular normalization may induce compensatory chemokine-mediated MDSC recruitment	Combine hypoxia modulation with CXCR2/CCR1/CCL-axis blockade	(26, 70)
CD8+ T cell exhaustion	Drives terminal exhaustion, CD39–adenosine signaling, mitochondrial stress	HIF-1α can be protective or harmful depending on activation state and chronicity	Preserve effector metabolism while reducing chronic hypoxic stress	(88)
NK cell dysfunction	Reduces cytotoxicity, synapse formation, and granzyme release	IL-2/PI3K–mTOR activation may restore HIF-dependent NK adaptation	Combine oxygen/metabolic modulation with NK-activating strategies	(35, 89)
Antigen presentation loss	Reduces MHC-I/MHC-II presentation and DC function	Checkpoint blockade may fail if antigen visibility is not restored	Combine ICB with strategies restoring MHC expression or DC activation	(90)
RNA modifications	m6A/ac4C regulate HIF translation, chemokines, myeloid metabolism, PD-L1	Current evidence is often correlative; site-specific causal evidence remains limited	Identify tumor- or immune-cell-specific RNA modification circuits	(8, 68)

## 6 Therapeutic opportunities and translational considerations

Given the multilayered nature of hypoxia-driven immune escape, therapeutic strategies should not be limited to a single pathway. Instead, rational intervention requires matching the dominant hypoxia-associated immune barrier with appropriate agents, biomarkers, and combination strategies. To better connect mechanistic insights with translational opportunities, **Table 2** summarizes representative therapeutic approaches targeting hypoxia-driven immune escape, including relevant agents or strategies, combinatorial rationale, potential biomarkers, and major translational challenges.

**Table 2:** Therapeutic strategies targeting hypoxia-driven tumor immune escape.

Strategy	Representative agents/approaches	Combination rationale	Potential biomarkers	Key barriers	Ref
HIF-2α inhibition	Belzutifan, PT2385/related agents	Reduce HIF-driven immune suppression; combine with anti-PD-1/PD-L1	HIF-2α, CAIX, VEGF, hypoxia signature	Tumor-type specificity; immune-cell effects	(73, 91)
HIF-1/HIF-2 inhibition	32-134D and related inhibitors	Reduce lactate, VEGF, TAM/MDSC recruitment	HIF signature, LDHA, GLUT1, PD-L1	Limited clinical validation	(92)
Hypoxia-activated prodrugs	Evofosfamide and related agents	Kill hypoxic tumor cells; release antigens; combine with ICB	Hypoxia imaging, necrotic/hypoxic fraction	Variable efficacy; patient selection	(93)
Anti-angiogenic/vascular normalization	VEGF/VEGFR inhibitors	Improve perfusion and immune infiltration; reduce hypoxia	VEGF, vascular signatures, hypoxia PET	Narrow normalization window	(94)
Lactate/acidosis targeting	LDHA/MCT inhibitors, buffering strategies	Restore T/NK function; reduce TAM/MDSC polarization	Lactate, LDHA, MCT1/4, pH signatures	May affect immune-cell metabolism	(95)
Adenosine-axis blockade	CD39/CD73/A2A receptor inhibitors	Reverse hypoxia-associated immunosuppression	CD39, CD73, adenosine signature	Redundant suppressive pathways	(96, 97)
Myeloid reprogramming	CSF1R, CCR2/CXCR2, PI3Kγ inhibitors	Reduce TAM/MDSC-mediated resistance	TAM/MDSC signatures, CCL2, CXCL5	Myeloid heterogeneity	(98–100)
RNA epigenetic targeting	ALKBH5, YTHDF2, NAT10-related strategies	Block hypoxia-dependent RNA regulatory circuits	m6A/ac4C regulators, target RNA signatures	Early stage; specificity and toxicity	(101–103)
Timing/sequencing strategy	Hypoxia modulation before or during ICB	Prime immune infiltration before checkpoint release	Dynamic hypoxia and immune biomarkers	Optimal schedule unknown	(29, 104)

### 6.1 Targeting hypoxia and HIF signaling

Because hypoxia is an upstream driver of immune escape, targeting HIF signaling is an attractive strategy (69). HIF inhibitors may act on tumor metabolism, angiogenesis, immune checkpoints, and immune cell recruitment at the same time. The dual HIF-1/HIF-2 inhibitor 32-134D suppresses tumor growth in hepatocellular carcinoma models (70). It also reduces TAMs and MDSCs while increasing CD8+ T cell and NK cell infiltration, helping convert cold tumors into hotter tumors (70). The HIF-2α inhibitor PT2385 reduces TAMs and Tregs in glioblastoma models and enhances anti-PD-1 efficacy (71). These studies suggest that HIF inhibition can reprogram the metabolism–immune–vascular axis. HIF inhibition may also reduce PD-L1 expression, suppress chemokine production such as CCL2 and CXCL5, relieve lactate-mediated suppression, enhance cGAS–STING activation, and improve antigen presentation (71). However, HIF targeting must be carefully optimized because HIF signaling can also support immune cell adaptation in some contexts.

Several hypoxia-related agents have entered clinical or late preclinical development, although their immunological applications remain incompletely defined. HIF-2α inhibitors, such as belzutifan, have demonstrated clinical activity in HIF-2α-driven tumors and provide proof-of-concept that hypoxia-response pathways can be therapeutically targeted in patients (72). Earlier HIF-2α inhibitors, including PT2385 and PT2977/belzutifan-related compounds, also support the feasibility of pharmacological HIF-2α blockade (72, 73). In addition, hypoxia-activated prodrugs, such as evofosfamide, and VEGF/VEGFR-targeted anti-angiogenic agents indirectly modulate hypoxic niches by altering oxygenation, vascular normalization, or hypoxia-selective cytotoxicity (74). However, most of these agents were not originally developed as immunotherapy sensitizers. Their optimal use in combination with immune checkpoint blockade therefore requires further mechanistic and clinical evaluation.

### 6.2 Combinatorial rationale with immunotherapy

HIF inhibition alone may not be sufficient because tumors can escape through parallel immunosuppressive pathways. Therefore, combining hypoxia targeting with immunotherapy is a logical strategy. In hepatocellular carcinoma models, 32-134D combined with anti-PD-1 therapy increases tumor clearance rates compared with single treatment (75). This effect is associated with reduced glycolysis and VEGF signaling, vascular normalization, decreased TAM and MDSC infiltration, and increased CD8+ T cell and NK cell activity (75). In post-ablation residual tumors, melatonin reduces HIF-1α and PD-L1 expression, decreases MDSCs, increases CD8+ T cell infiltration, and improves anti-PD-L1 therapy (60). In cell therapy, metabolic preconditioning through AMPK activation and mTOR inhibition can improve CAR-T mitochondrial fitness, survival, and cytotoxicity in hypoxic tumors (76). Epigenetic pathways also matter. HIF-1α can cooperate with HDAC1 and PRC2 to suppress effector gene expression in T and NK cells, and targeting this axis may overcome PD-1 resistance (77). These findings support a broader principle: hypoxia-targeted therapy should not only reduce oxygen stress but also restore immune function. The best combinations may depend on whether the dominant barrier is myeloid suppression, T cell exhaustion, poor antigen presentation, high lactate, or checkpoint activation.

The rationale for combining hypoxia-targeted therapy with immunotherapy is based on the fact that hypoxia suppresses antitumor immunity at multiple nonredundant levels. First, hypoxia induces PD-L1 expression and adenosine signaling, providing a direct rationale for combining HIF or adenosine-axis blockade with anti-PD-1/PD-L1 therapy (9). Second, hypoxia promotes lactate accumulation, acidosis, and glucose competition, suggesting that metabolic intervention may improve T-cell and NK-cell fitness before or during checkpoint blockade (9). Third, hypoxia recruits TAMs, MDSCs, and Tregs, supporting combinations with myeloid-reprogramming agents, CXCR2/CCR2 inhibitors, CSF1R blockade, or Treg-targeting strategies. Fourth, hypoxia impairs antigen presentation, indicating that checkpoint blockade may be more effective when combined with approaches that restore MHC expression, dendritic cell activation, or innate immune sensing. Therefore, the most effective combination should be selected according to the dominant hypoxia-driven immune barrier in each tumor rather than applying a single universal regimen.

### 6.3 Targeting noncanonical hypoxia stress pathways

Because hypoxia activates multiple stress-response pathways beyond HIF, therapeutic strategies should not be restricted to direct HIF inhibition. Targeting ER stress or the PERK–ATF4 pathway may help restore antigen presentation and reduce T-cell dysfunction in selected contexts. Modulating autophagy may enhance antigen visibility or sensitize tumor cells to immune-mediated killing, although this approach requires caution because autophagy can also support immune cell survival. Mitochondrial protective strategies, ROS modulation, and metabolic fitness enhancement may improve the persistence and cytotoxicity of T cells, NK cells, and CAR-T cells in hypoxic tumors (78). These approaches may be especially useful when HIF inhibitors alone fail to reverse immune suppression. Future combination strategies should be designed according to the dominant hypoxia-associated stress program in each tumor, such as HIF activation, ER stress, mitochondrial dysfunction, ROS accumulation, or autophagy-dependent antigen loss.

### 6.4 NA epigenetics-based immunotherapy

RNA epitranscriptomic regulation provides new therapeutic opportunities. In glioblastoma, targeting ALKBH5 reduces NEAT1-dependent CXCL8 expression, lowers TAM recruitment, and restores CD8+ T cell function (67). In MDSCs, blocking the HIF-1α–YTHDF2–PFKL axis may reduce glycolysis and suppressive activity (55).NAT10 inhibition reduces HIF1A translation, lactate accumulation, PD-L1 expression, and immune suppression, improving anti-PD-L1 response (79). m6A readers such as YTHDF1 and YTHDF2 may also regulate antigen processing, chemokine production, and MDSC recruitment (80, 81). This places RNA modification at the intersection of metabolism, immune suppression, and antigen presentation. However, RNA modification-based immunotherapy is still in an early stage. These enzymes regulate many transcripts in normal and tumor cells. Broad inhibition may cause toxicity or unexpected immune effects. A more precise future direction is to identify hypoxia-dependent RNA modification circuits that are selectively required in tumors or suppressive immune cells.

### 6.5 Biomarker-guided patient selection

Biomarker-guided patient selection will be essential for translating hypoxia-targeted immunotherapy. Potential biomarkers include hypoxia gene signatures, HIF-1α or HIF-2α expression, CAIX, GLUT1, LDHA, VEGF, lactate-related metabolic signatures, adenosine-pathway markers such as CD39 and CD73, and immune checkpoint expression such as PD-L1 (82). Spatial biomarkers may be particularly informative because hypoxia is heterogeneous within tumors. For example, co-localization of hypoxic regions with TAMs, MDSCs, Tregs, or exhausted CD8+ T cells may identify patients more likely to benefit from hypoxia–immune combination therapy (83). Imaging-based approaches, including hypoxia PET tracers, may also help define tumor hypoxia burden and monitor dynamic changes during treatment. In the future, integrated biomarkers combining hypoxia status, immune infiltration, metabolic state, and RNA modification signatures may guide patient selection and treatment stratification.

### 6.6 Timing and sequencing considerations

The timing and sequencing of hypoxia-targeted therapy with immunotherapy may strongly influence therapeutic outcomes. Hypoxia modulation before immune checkpoint blockade may improve vascular function, reduce lactate and adenosine accumulation, enhance antigen presentation, and increase immune cell infiltration, thereby converting an immune-cold tumor into a more responsive state. In contrast, concurrent treatment may be preferable when hypoxia rapidly induces PD-L1 expression, myeloid recruitment, or T-cell exhaustion during therapy. Sequential approaches may also reduce toxicity by allowing transient vascular normalization or metabolic reprogramming before immune activation (1). However, prolonged or excessive inhibition of hypoxia-response pathways may impair the metabolic adaptation of activated T cells and NK cells. Therefore, future studies should define optimal therapeutic windows, dosing schedules, and sequence-dependent immune effects rather than simply combining agents at maximum tolerated doses.

### 6.7 Translational barriers and safety considerations

Several translational barriers should be considered. First, hypoxia is spatially and temporally heterogeneous, making it difficult to select patients and monitor treatment response using single-site biopsies (1). Second, HIF signaling and metabolic adaptation may have opposite roles in tumor cells and immune cells, raising concerns that systemic pathway inhibition could suppress antitumor immunity in some contexts. Third, metabolic interventions targeting glycolysis, lactate transport, adenosine signaling, or mitochondrial function may also affect activated lymphocytes, NK cells, and dendritic cells (1). Fourth, RNA modification enzymes regulate many transcripts in both tumor and normal tissues, so broad inhibition may cause toxicity or unpredictable immune effects. Finally, hypoxia-targeted combinations may increase overlapping toxicities with immunotherapy, anti-angiogenic therapy, radiotherapy, or chemotherapy (1). These challenges highlight the need for rational dosing, patient stratification, pharmacodynamic biomarkers, and early-phase trials designed to evaluate immune remodeling rather than tumor response alone.

## 7 Discussion and perspectives

Hypoxia is a defining feature of the tumor microenvironment and a central driver of immune escape. Through HIF-1α and HIF-2α, hypoxia integrates metabolic reprogramming, immune checkpoint activation, myeloid suppression, T cell exhaustion, Treg stability, NK dysfunction, and impaired antigen presentation. As a result, tumors shift from immune-active states toward immunosuppressive and therapy-resistant states. A major lesson from current studies is that hypoxia does not act through a single pathway. It creates a multi-layered immune barrier. This barrier includes lactate accumulation, acidosis, PD-L1 induction, TAM and MDSC expansion, Treg enrichment, CD39–adenosine signaling, antigen presentation loss, and RNA modification-dependent post-transcriptional control. These processes reinforce one another, which explains why single-agent approaches often show limited activity.

Despite strong evidence linking hypoxia to tumor immune escape, several conceptual challenges remain unresolved. First, hypoxia is often discussed as a uniformly immunosuppressive condition, but its effects differ between tumor cells, myeloid cells, T cells, NK cells, and dendritic cells (1). Second, HIF signaling is not always detrimental to antitumor immunity, because it may support short-term metabolic adaptation in activated lymphocytes while promoting exhaustion under chronic stress (1). Third, many therapeutic strategies targeting hypoxia have shown context-dependent efficacy, suggesting that hypoxia is not a single targetable pathway but a composite state involving oxygen deprivation, metabolic competition, acidosis, vascular dysfunction, and stress-response signaling. These issues underscore the need to move from pathway-based targeting to context-guided combination strategies.

Future research should focus on several priorities. First, single-cell, spatial transcriptomic, metabolomic, and epigenomic approaches are needed to map the spatial heterogeneity of hypoxic immune niches. Second, therapies should be designed based on the dominant hypoxia-driven resistance mechanism in each tumor. Some tumors may require HIF inhibition, while others may benefit more from lactate targeting, myeloid reprogramming, checkpoint blockade, or RNA modification targeting. Third, RNA epitranscriptomic regulation should be studied as a functional layer, not only as a biomarker. Site-specific and cell-type-specific validation will be essential. Overall, effective therapy will likely require multi-layered targeting of the hypoxia–metabolism–immune axis. By combining hypoxia modulation, metabolic intervention, immune checkpoint blockade, cell therapy optimization, and RNA modification targeting, it may be possible to reverse tumor immune escape and improve durable responses to cancer immunotherapy. From a translational perspective, future studies should prioritize clinical-stage agents, biomarker-defined patient populations, pharmacodynamic monitoring of hypoxia and immune remodeling, and rational timing or sequencing with immune checkpoint blockade.
